# Predictors of return to work among patients in treatment for common mental disorders: a pre-post study

**DOI:** 10.1186/s12889-017-4581-4

**Published:** 2017-07-18

**Authors:** Mattias Victor, Bjørn Lau, Torleif Ruud

**Affiliations:** 1 Lovisenberg Hospital, Postboks 4970, Nydalen, 0440 Oslo, Norway; 2 University of Oslo, Institute of Clinical Medicine, Oslo, Norway; 3 Department of Psychology, University of Oslo, Oslo, Norway; 4 Division Mental Health Services, Akershus University Hospital, 1478 Lørenskog, Norway

**Keywords:** Return to work, Common mental disorders, Work disability prevention, Sick leave, Sickness absence, Psychotherapy

## Abstract

**Background:**

Effects of return to work (RTW) interventions vary, and more knowledge is needed about the factors that contribute to RTW. This study investigated changes in work participation and mental health, and predictors of RTW among patients being treated for common mental disorders (CMDs).

**Methods:**

The study was a prospective pre–post study of 164 patients treated at an RTW outpatient clinic for CMDs. Differences between before and after treatment were analysed using paired *t* tests for continuous variables and marginal homogeneity test for categorical variables. Univariable and multivariable logistic regression analyses were used to identify factors associated with RTW. Baseline data (patient characteristics, clinical status, generalized self-efficacy, expectations of future work ability) and treatment variables were used as independent variables in logistic regressions. Further analysis investigated whether improvements in symptoms, work ability, expectations of future work ability and generalized self-efficacy were associated with RTW.

**Results:**

Number of individuals with full work participation increased, and there were improvements in symptoms, work ability and generalized self-efficacy. In the final model for predicting RTW, baseline work ability and expectancy of future work ability, a history of psychiatric treatment and focus on RTW in the treatment predicted RTW. Improvement in expectations of future work ability at post-treatment did also predict RTW.

**Conclusions:**

Assessing work ability and expectations of RTW at the beginning of treatment is recommended to identify patients at risk of long-term sick leave. Individuals with a history of psychiatric treatment are also risking long-term work disability. It is essential that treatment focus not only on symptom-relief, but also on improving work ability and expectations of RTW. An RTW-focused approach in therapy is associated with RTW.

**Trail registration:**

ClinicalTrails.gov ID NCT01181635. Registered 08/12/2010.

**Electronic supplementary material:**

The online version of this article (doi:10.1186/s12889-017-4581-4) contains supplementary material, which is available to authorized users.

## Background

Mental problems are a large and increasingly frequent reason for sickness absence and disability pension in the Western world and contribute to huge economic and quality of life-related losses for both society and individuals. Common mental disorders (CMDs), such as depression and anxiety, are the main contributors to the economic burden of reduced workdays [1]. In Norway, CMDs account for about one-fifth of sick leave episodes and one-third of all disability pensions [2]. Programmes and interventions have in recent years been developed to facilitate return to work (RTW) after sickness absence because of mental disorders [3]. The effects of these interventions vary. A recent meta-analysis of 16 randomized controlled studies found that the available interventions did not improve the total number of individuals with successful RTW, but did reduce the time until RTW in the intervention group [4]. Cognitive behavioural therapy (CBT) and problem-solving therapy have reduced sick leave in some studies [4–6]. There is also some evidence that early interventions [3] can support RTW.

RTW after sickness absence is a multifactorial process, and health-related factors only partly explain individual differences [7, 8]. Many variables have been identified as predictors of RTW, but the research is inconclusive. Older age is often found to be a predictor of long-term disability among persons sick-listed because of mental disorders, but research findings vary regarding other personal factors (sex, education, marital status) [8, 9]. Depression and anxiety [8–10], duration and severity of depressive symptoms [11], duration of sickness absence at the onset and earlier episodes of sickness absence [12] are associated with RTW. A person’s own expectations of RTW is a predictor of actual RTW [13, 14]. Self-efficacy, a person’s belief in being able to handle new and challenging situations, also predicts RTW [15]. Working environment is also associated with sickness absence and RTW [16, 17]. Present knowledge about predictors of RTW largely comes from cohort studies analysing baseline data as potential prognostic factors. There is a lack of interventional studies that combine baseline data with treatment variables in analyses of predictors for RTW. A better understanding of predictors for RTW among patients in treatment for CMDs could help to identify individuals in risk of prolonged RTW, and possibly to make interventions more efficient.

In this paper we wanted to investigate which factors are associated with RTW among patients in treatment for CMDs. We wanted to use both baseline data, information on treatment and changes in symptoms and functioning during treatment in analyses of predictors for RTW. Specifically, we wanted to address the following questions: a) Are there changes in work participation, work ability, expectations of future work ability, generalized self-efficacy and symptoms between pre- and post-treatment? b) Which patient and treatment factors are associated with RTW? and c) Are improvements in symptoms, work ability, expectations of future work ability and generalized self-efficacy associated with RTW?

## Methods

### Design and treatment setting

A prospective pre–post study design was used to investigate changes in work participation, work ability, expectations of future work ability, generalized self-efficacy and symptoms in patients treated at an RTW outpatient clinic. Changes in work participation was used to identify cases with successful RTW. Patient characteristics and clinical status at baseline, as well as treatment variables and changes on clinical measures were then used to investigate predictors for RTW. This study was part of a larger study that also investigated patient characteristics at baseline [18]. Data were collected from medical records and from patients and therapists using paper- and pencil questionnaires.

The RTW clinic is part of the Lovisenberg Community Mental Health Centre in Oslo, and employs clinical psychologists as therapists to treat patients referred by their general practitioners (GPs). To be included in the RTW programme, a patient must have a job and be entitled to sick leave benefits. In Norway, a person can receive sick leave benefits for a maximum of 52 weeks; after that, the person may apply for other types of benefits. Patients in the RTW programme can either be on sick leave already or at risk of requiring sick leave (according to their general practitioners) because of mental health problems. The programme therefore also has a preventive function. The treatment offered is individual, time-limited psychotherapy and/or psycho-educative courses for various problems such as depression, social phobia, panic disorder, stress and insomnia. No changes in treatment procedures were introduced as part of this study.

### Procedures and participants

**Fig. 1:**
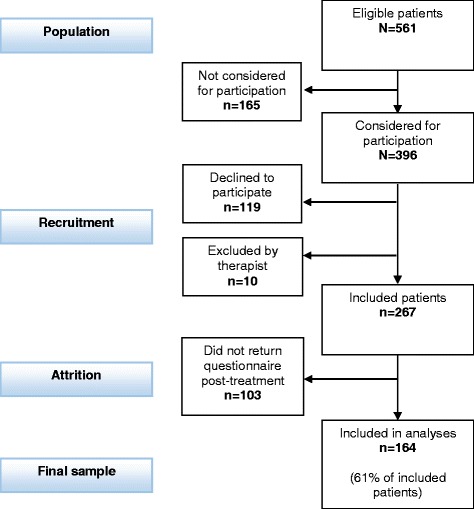
Flowchart of patient recruitment

Participants were recruited from a population of 561 patients who started their treatment during 10 consecutive months (August 16th, 2010, to June 15th, 2011). Figure 1 shows the recruitment process. All new patients were eligible for participation in the study. One hundred and sixty-five of the eligible patients were never asked to participate, primarily because the therapists forgot to ask the patients to participate or because the therapists misunderstood which patients should be asked. One hundred and nineteen patients declined to participate, and ten patients were excluded. Reasons for exclusion were that the therapist considered that participation would be a burden for the patients (*n* = 8), or because patients were not sufficiently fluent in the Norwegian language to complete the questionnaire (*n* = 2). Of the 267 included patients, 103 did not return the questionnaire at the end of treatment. The final sample therefore comprised 164 patients who completed the questionnaire at both pre- and post-treatment.

To investigate whether recruitment had introduced a selection bias, we examined whether there were differences between included patients (*n* = 267) and all other eligible patients (*n* = 294). Data were collected from medical records for all patients in the population. No statistically significant differences were found for age, sex, therapist-reported scores of functioning and symptoms, or psychiatric diagnoses. Therefore, we concluded that included patients did not differ significantly from the population on any of these variables. To determine whether dropout had introduced a bias in the final sample, we examined whether there were differences between the 164 patients who returned the questionnaire at both the beginning and the end of treatment and the 103 patients who answered only at the beginning. These analyses are not shown, but no statistically significant differences were found for age, sex, marital status, education, history of psychiatric treatment, aspects of work situation causing present problem or diagnoses, or for baseline scores of patient-reported symptoms, work ability, self-efficacy and expectancy of future work ability. Furthermore, there were no statistically significant differences in treatment modality, number of sessions or time on a waiting list. We therefore concluded that the sample did not differ significantly from all included patients for those variables that could be checked.

The patient characteristics at baseline are shown in Table 1. Their mean age was 38.2 years (standard deviation (SD) = 10.4); 69% had a college or higher university degree. Most patients had a CMD. Eighteen patients (11%) met only for psycho-educative courses and were never diagnosed, and these patients were excluded from the analyses of the diagnoses. Ninety-six patients (59%) reported that aspects of their at work situation were causing the problems for which they were seeking help. One hundred and thirty patients (81%) scored above the clinical cut-off for psychological distress at baseline.

**Table 1:** Patient characteristics at the baseline^a^

Patient characteristic		N	%
Age	18–29	33	20
	30–39	69	42
	40–49	35	21
	50–	27	17
Sex	Women	116	71
	Men	48	29
Marital status	Living with partner	84	52
	Living alone	79	49
Education	Comprehensive school (1–9 yr)	11	7
	Secondary/vocational school (10–12 yr)	40	25
	College degree (13–16 yr)	78	48
	Higher university degree (>16 yr)	34	21
Main diagnosis (ICD-10)	Depression (F32-F33)	65	45
	Anxiety (F40-F42)	31	21
	Adjustment disorder (F43)	34	23
	Other psychiatric diagnoses	12	8
	Z-diagnoses	4	3
History of psychiatric treatment	Yes	80	49
	No	84	51
Aspects of work situation causing present problem	1) Yes, definitely	34	21
	2) Yes, to some degree	62	38
	3) No, not really	37	23
	4) No, absolutely not	30	18

^a^N varies between 146 and 164 due to missing cases

### Measurements

The questionnaire to the patients covered socio-demographics (age, sex, marital status and educational level), work situation and mental health [see Additional file 1]. In the logistic regression analyses, a dichotomous variable was created for educational level (more/less or equal than 13 years of education). The therapists diagnosed the patients and completed a form containing questions covering each patient’s work situation and treatment history [see Additional file 2]. After the treatment, the therapists answered questions covering the treatment content and the patient’s present status [see Additional file 3]. Information about treatment modality, number of sessions and waiting time was collected from the patients’ medical records.

#### Work participation and RTW

Based on information from the questionnaires filled in by the patients and therapists, and medical records, an index for work participation was constructed with three mutually exclusive categories: 1) *Working fully*; 2) *Working partly* (worked part-time and were either on sick leave, receiving a social benefit or partially unemployed); 3) *Not working* (full sick leave or unemployed and/or receiving social benefits, or studying full time, including working additional hours part-time or receiving some form of social benefit).

Using the categories for work participation at pre- and post-treatment, a new variable with five categories to define RTW was created. Moving to Working fully at post-treatment from any of the other categories at pre-treatment was defined as *Full RTW*. Moving to Working partly from not working at pre-treatment was defined as *Partial RTW*. No change in work participation was defined as either *Still working fully*, *Still working partly* or *Still not working*. Moving to a category indicating lower work participation was defined as *Working less*. In the logistic regression analyses, a dichotomous variable for RTW was created as follows. Participants classified as having full or partial RTW were clustered together with those still working fully or partly, and their cases were defined as *successful RTW*. Those working less were clustered together with those still not working, and their cases were defined as *failed RTW*.

#### Mental health and psychological variables

Patient-reported symptoms were measured using the Clinical Outcomes in Routine Evaluation Outcome Measure (CORE-OM) [19]. The CORE-OM comprises 34 items related to the previous week that address four domains: problems, functioning, subjective well-being and risk. All items are scored from Never (= 0) to Almost all the time (= 4). Total mean scores are usually multiplied by 10 before being presented as a total score ranging from 0 to 40 [20]. Forms with fewer than 90% of the items completed were excluded from the analyses of the total score. Evans et al. have reported an internal consistency for the CORE-OM of Cronbach’s coefficient (α) = 0.94 and a 1-week test–retest reliability of Spearman’s *r* = 0.90 [21]. In line with Evans et al., we found a Cronbach’s α = 0.92 in this study, using baseline data. In the logistic regression analyses, the total score and the problem subscale scores for depression and anxiety were used.

The therapists diagnosed the patients according to the International Classification of Diseases 10 (ICD-10) guidelines [22], and the ICD-10 diagnoses were clustered into five categories: *Depression* including depressive episodes (F32) and recurrent depressive episodes (F33), *Anxiety* including phobic anxiety disorders (F40), other anxiety disorders (F41) and obsessive–compulsive disorder (F42), *Adjustment disorders* (F43), *Other psychiatric diagnoses* (e.g., substance abuse and eating disorders); and 4) *Z-diagnoses* (reasons for contact with health services not resulting in a psychiatric diagnoses, e.g., examination). For the logistic regression analyses, three dummy variables were created, in which each of the diagnoses Depression, Anxiety and Adjustment disorder was contrasted against the other two combined diagnoses.

The work ability index (WAI) is a seven-item questionnaire used in occupational health services and research to assess work ability [23]. We used two single items from the WAI. The first item, “*Assume that your work ability at its best has a value of 10 points. How many points would you give your current work ability?*” is scored from 0 (= worse) to 10 (= best). A high correlation has been found between this single item to rate work ability and the total WAI score and, therefore, the single item is often used instead of the complete instrument [24]. The second question is “*Do you believe, according to your present state of health, that you will be able to do your current job two years from now?*” The original alternative answers in this question are: 1) Unlikely, 2) Not certain and 3) Relatively certain. To make it possible to dichotomize the answers, we instead used the following answer alternatives: 1) Yes, definitely, 2) Yes, to some degree, 3) No, not really and 4) No, absolutely not. In the logistic regression analysis of predictors of RTW, a dichotomous variable was made by merging the first two alternatives and the last two alternatives. A dichotomous variable was also made for change on this variable: answers indicating either a change from a negative to a positive expectancy of future work ability, or a maintained positive expectancy of future work ability were combined, while answers indicating either a change from a positive to a negative expectancy of future work ability, or a maintained negative expectancy of future work ability were combined. Patients also indicated to what extent the problems they were seeking help for were caused by aspects of their work situation, using the following answer alternatives: 1) Yes, definitely, 2) Yes, to some degree, 3) No, not really and 4) No, absolutely not. In the logistic regression analysis of predictors of RTW, a dichotomous variable was made by merging the first two alternatives and the last two alternatives.

The Generalized Self-Efficacy Scale (GSE) assesses an individual’s beliefs in his or her own ability to deal with new or difficult situations [25]. Possible responses on the 10 items range from Not at all true (= 1) to Exactly true (= 4). A mean score of 1 to 4 is calculated for all items. The scale has been used in many research projects, and typically yields an internal consistency of Cronbach’s α 0.75 to 0.91 [26]. Using baseline data, we found a Cronbach’s α = 0.90 in this study.

#### Treatment variables

The therapeutic interventions were categorized as *individual psychotherapy*, *group intervention* or *mixed intervention* (both group and individual treatment). One hundred and nine (67%) patients received individual psychotherapy, 21 (13%) received group interventions and 34 (21%) received combined treatment. For the logistic regression analyses, three dummy variables were created, in which each treatment modality was contrasted against the other two modalities combined. The number of sessions and time from referral to the first session were recorded. The average number of sessions were 20 (range 1–96) for the entire sample, and 18, 12 and 33 for patients who received individual psychotherapy, group interventions and combined treatment, respectively. The average time on a waiting list was 51 days (range 2–172).

At the end of the treatment, the therapists reported on a scale of 1–9 how much the intervention had focused on RTW. The Therapist Interventions and Qualities Inventory (TIQI) [27]) was used to assess the content of each individual therapy. The TIQI has 36 items that cover the most commonly used therapeutic interventions in addition to therapist qualities such as empathy. The TIQI statements are rated from Not at all (= 0) to Very much (= 4). Factor analysis has yielded eight factors: Empathic therapist, Transference focus, Practical advice, Cognitive–behavioural, Care and support, Psychodynamic approach, Medical approach and Therapist frustrated [27]. A mean for each factor of 0–4 was generated to indicate how much that factor was present in each treatment. The mean for each factor was used as an independent variable in the logistic regression analyses.

### Statistical analyses

Data were analysed using SPSS for Windows (version 24, IBM Corp., Armonk, NY). Differences between before and after treatment were analysed using paired *t* tests for continuous variables and marginal homogeneity test for categorical variables. In all analysis of differences between groups, a statistical significance level of *p* < 0.05 was used. Effect sizes (Cohen’s d) were calculated for changes in work ability, generalized self-efficacy and total score on the CORE-OM, and were defined as small (d = 0.2), medium (d = 0.5) or large (d = 0.8) [28]. We used the model of reliable and significant change to analyse changes in the CORE-OM scores [29, 30]. This model comprises two steps: firstly, calculating a criterion for identifying a reliable change using the psychometric properties of the test and secondly establishing a clinical cut-off using the SD from a nonclinical sample. It is recommended that the measure of internal consistency be used when calculating the criteria for identifying a reliable change in clinical populations [31]. The use of the Cronbach’s coefficient (α) found in this study, α = 0.92, produced a criterion for reliable change of 4.20. We adopted Jacobson and Truax’s criterion c for establishing the clinical cut-off. Using the SD from a Norwegian nonclinical sample [32] resulted in a clinical cut-off of 11.02. Cases with a reliable reduction of CORE-OM scores were defined as *Improved*, and cases with reliable increases were defined as *Worse*. Cases with a reliable change from above the cut-off to below the cut-off were defined as *Recovered*. Logistic regression analysis was used to identify factors associated with RTW. Univariable logistic regression analyses were first performed for all independent variables, with RTW as dependant variable. Variables that had a *p*-value of <0.15 in the univariable analyses were selected for inclusion in the multivariable logistic regression analysis. Multicollinearity between the remaining independent variables was tested by checking the variance influence factor (VIF) statistics. Multicollinearity was assumed when VIF scores were >4. A multivariable logistic regression analysis with manual backwards stepwise selection was then performed, using a *p*-value of <0.05 as cut-off for the inclusion in the final combined model (Wald statistics).

## Results

### Changes during treatment

Changes in work participation and clinical outcome measures are shown in Table 2. There were statistically significant changes on all outcome measures. Of those working fully at pre-treatment, 58 (83%) had maintained full work participation at the end of treatment, and 12 (17%) were working less. Of those working partly, 18 (45%) achieved full RTW, 17 (43%) had maintained partial work participation and 5 (13%) were working less. Of those not working at pre-treatment, 18 (33%) achieved full and six (11%) partial RTW, and 30 (56%) were still not working. Participants with either full (*n* = 36, 22%) or partial RTW (*n* = 6, 4%), or full (*n* = 58, 35%) or partially (*n* = 17, 10%) maintained work participation were defined as *successful RTW* (*n* = 117, 71%). Thirty patients (18%) were not working at either pre- or post-treatment, and 17 (10%) were working less at post-treatment than at pre-treatment. These 47 (29%) cases were defined as *failed RTW*.

**Table 2:** Outcome measures. Changes between pre- and post-treatment in work participation, expectations of future work ability, workability, generalized self-efficacy and symptoms^a^

	Pre-treatment	Post-treatment	Significance testing	
	N (%)	N (%)	t-value	Sig.
Work participation				0.001^b^
Fully working	70 (43)	94 (57)		
Partially working	40 (24)	29 (18)		
Not working	54 (33)	41 (25)		
Expectations about future work ability (2 yr. from now)				0.004^b^
Yes, definitely	58 (48)	77 (64)		
Yes, to some degree	40 (33)	30 (25)		
No, not really	21 (17)	10 (8)		
No, absolutely not	2 (2)	4 (3)		
	Mean (SD)	Mean (SD)		
Work ability	4.7 (2.7)	6.8 (2.6)	−8.76	<0.001
Generalized self-efficacy	2.6 (0.5)	2.8 (0.5)	−5.96	<0.001
CORE-OM Total	16.5 (5.4)	10.7 (5.5)	14.02	<0.001
CORE-OM Anxiety	19.3 (8.3)	11.6 (7.9)	11.38	<0.001
CORE-OM Depression	22.5 (8.9)	13.4 (8.4)	12.87	<0.001

^a^N varies between 121 and 164 due to missing cases

^b^Marginal homogeneity test, asymptotic sig. (2-sided)

After treatment, 19 (33%) more patients had the highest level of expectations of being able to work in 2-years’ time. The number of patients with negative expectations of being able to work in 2-years’ time was reduced by 9 (39%). Self-assessed work ability increased by 2.0 points (43%). Analyses of changes in the group mean showed significant changes on all clinical and psychological outcome measurements, which yielded effect sizes of d = 0.4 for generalized self-efficacy, d = 0.8 for work ability and d = 1.1 for reduction of the total CORE-OM score. When using the criteria for reliable and significant change as shown in the statistical analyses section, 59 (37%) patients were considered to have recovered, and 32 (20%) to have improved; two (1%) patients became worse.

### Predictors of RTW

The results of the logistic regression analysis to identify factors associated with RTW are shown in Table 3. In the univariable analyses the following variables had an association with RTW of *p* < 0.15: history of psychiatric treatment, total CORE-Om score, CORE-OM depression score, work ability, generalized self-efficacy, expectancy of future work ability, focus on RTW and cognitive behavioural therapy. Analysis of VIF statistics showed multicollinearity between total CORE-Om score and CORE-OM depression score (VIF > 4), and therefore only total CORE-Om score were included in the multivariable analysis. A backwards stepwise multivariable logic regression analysis was then performed with *p* < 0.05 as cut-off for including independent variables in the final model. This resulted in a final model for predicting RTW, with history of psychiatric treatment, work ability, expectancy of future work ability and focus on RTW as predictors. The strongest positive predictor of RTW was a pre-treatment positive expectancy of future work ability, with an odds ratio (OR) of 4.50. Having a history of psychiatric treatment was a negative predictor for RTW, with an OR of 0.36.

**Table 3:** Univariable and multivariable associations with RTW^a^

	Univariable associations				Multivariable associations in the final model			
	OR	95% CI		*p*	OR	95% CI		*p*
Patient characteristics at baseline		Lower	Higher			Lower	Higher	
Sex (women)	0.66	0.30	1.44	0.297				
Age, yr. (older)	0.99	0.95	1.02	0.402				
Marital status (living with partner)	1.16	0.59	2.28	0.673				
Education (≥13 yr)	1.19	0.58	2.46	0.629				
History of psychiatric treatment (yes)	0.48	0.24	0.96	0.038^c^	0.36	0.15	0.86	0.021
Mental health at baseline								
Diagnosis of depression	0.80	0.38	1.70	0.566				
Diagnosis of anxiety	1.31	0.53	3.26	0.560				
Diagnosis of adjustment disorder	1.04	0.44	2.45	0.931				
Higher CORE-OM total	0.92	0.86	0.99	0.018^c^				
Higher CORE-OM Depression	0.97	0.93	1.01	0.106				
Higher CORE-OM Anxiety	1.02	0.98	1.06	0.313				
Higher work ability	1.24	1.08	1.42	0.002^c^	1.21	1.03	1.43	0.021
Higher generalized self-efficacy	2.29	1.17	4.50	0.016^c^				
Positive expectancy of future work ability (yes)	4.60	2.00	10.57	<0.001^c^	4.50	1.64	12.34	0.003
Aspects of work situation causing present problem (yes)	0.75	0.37	1.51	0.416				
Treatment variables								
Individual therapy	0.68	0.33	1.43	0.314				
Group intervention	1.83	0.58	5.75	0.303				
Combined intervention	1.15	0.49	2.69	0.751				
More sessions	0.98	0.96	1.01	0.155				
Longer time on waiting list	1.00	0.99	1.01	0.563				
Greater focus on RTW	1.17	0.98	1.38	0.081^c^	1.23	1.02	1.49	0.034
Empathic therapist^b^	0.75	0.32	1.74	0.503				
Transference focus^b^	0.77	0.44	1.35	0.359				
Practical advice^b^	0.93	0.46	1.87	0.832				
Cognitive behavioural therapy^b^	1.85	1.09	3.14	0.022^c^				
Care and support^b^	0.85	0.47	1.55	0.593				
Psychodynamic approach^b^	0.85	0.48	1.52	0.594				
Medical approach^b^	0.71	0.30	1.68	0.434				
Therapist frustrated^b^	1.03	0.50	2.12	0.937				

Logistic regression analyses. The dependent variable was RTW; an odds ratio (OR) for RTW of >1 indicates a higher probability of successful RTW

^a^N varies between 128 and 164 due to missing cases

^b^Higher score

^c^These variables were included in a backwards stepwise multivariable logistic regression analysis resulting in the final model for predicting RTW

### Secondary outcome measures and RTW

Univariable logistic regression was performed with RTW as a dependent variable and changes on other outcome measures as independent variables (Table 4). Improved expectancy of future work ability, improved work ability and improved total CORE-Om score were associated with RTW with *p*-values <0.15. These three variables were therefore included in a backwards stepwise multivariable logistic regression with *p* < 0.05 as cut-off for including independent variables in the final model. This resulted in a final model in which only improved expectancy of future work ability was significantly associated with RTW, with an OR of 5.90.

**Table 4:** Logistic regression analyses to investigate whether changes in secondary outcome measures are associated with RTW^a^

	Univariable associations				Multivariable associations			
	OR	95% CI		*p*	OR	95% CI		*p*
Changes on secondary outcome measures		Lower	Higher			Lower	Higher	
Improved CORE-OM total	1.06	0.99	1.14	0.099^c^				
Improved work ability	1.16	1.03	1.32	0.019^c^				
Improved generalized self-efficacy	0.99	0.47	2.09	0.981				
Improved expectancy of future work ability (yes)^b^	5.90	1.81	19.24	0.003^c^	5.90	1.81	19.24	0.003

The dependent variable was RTW; an odds ratio (OR) for RTW of >1 indicates a higher probability of successful RTW

^a^N varies between 121 and 164 due to missing cases

^b^On this variable, being a case indicates either a change from negative to positive expectancy of future workability, or a maintained positive expectancy of future workability

^c^These variables were included in a backwards stepwise multivariable logistic regression with *p* < 0.05 as cut-off for including independent variables in the final model. This resulted in a final model in which only improved expectancy of future work ability was statistically significant associated with RTW

## Discussion

In this study, we investigated patient characteristics, clinical status, expectations of future work ability, generalized self-efficacy and treatment variables as predictors for RTW among patients in treatment for CMDs. The main finding is that positive expectations of future work ability, higher work ability at baseline and greater focus on RTW in the treatment were positive predictors for RTW, while having a history of psychiatric treatment was a negative predictor for RTW. In addition we found that if expectations of future work ability had improved after treatment, this was also a positive predictor for RTW.

### Changes during treatment

In this study, we found that 83% of patients at risk of needing sick leave continued working and that 49% of patients on full sick leave achieved full or partial RTW. These findings are consistent with those of other studies. Løvik et al. found that 79% of people at risk of going on sick leave had maintained work participation at the 6-month follow-up and that 54% of those on sick leave had achieved RTW [14].

Thirty-seven per cent achieved symptom recovery, and another 20% showed improvement of their symptoms. We found large effect sizes on improvements in other outcomes such as symptom reduction and increased work ability. Because our study did not include a control group, we could not test the effectiveness of the treatment. Self-assessed work ability, as measured by a single item in the WAI, increased by 2.0 points. This single item has been shown to be a strong predictor of future sick leave [24]. Sell found that a 1-point decrease in this item at the baseline was associated with a 15% increased risk of long-term sickness absence and a 33% increased risk of early retirement from the labour market [33]. We also found that generalized self-efficacy increased significantly, although there was a small effect size, which indicates a smaller change compared with the changes in the other secondary outcome measures. However, because self-efficacy is a trait-like quality, it can be argued that it is not expected to change greatly compared with measures of symptoms or states.

### Predictors of RTW

In the final model we found that positive expectations of future work ability and higher work ability at baseline, and greater focus on RTW in the treatment were positive predictors for RTW, while having a history of psychiatric treatment was a negative predictor for RTW. These four variables were associated with RTW also when controlled for symptom-score at baseline. This is in line with other studies showing that disorder-related factors are not the main predictors for sustained RTW [34]. The strongest positive predictor for RTW in our study was the individual’s own expectations of future work ability. This finding is consistent with the recent literature, which has found that expectations of future work ability and RTW are strong predictors of actual RTW [13, 14]. The only negative predictor for RTW in our final model was a history of psychiatric treatment. In our material this was a stronger predictor than present diagnosis and symptom-score at baseline. Having a history of psychiatric treatment is an indicator of recurrent mental health problems. It is possible that recurrent mental health problems have an impact on psychological factors like self-efficacy, which was a significant predictor in the uncontrolled analysis but did not make it into the final model. We used a measure of generalized self-efficacy. It is probable that a work-specific self-efficacy measure would be an even stronger predictor of RTW, and such an instrument has been developed recently [35]. Using this instrument, Nieuwenhuijsen et al. found that work self-efficacy predicted RTW even better that expectations of future work ability [15].

Of the treatment variables assessed in this study, only focus on RTW in the treatment was included in the final model. It is important to stress that this indicates an association with RTW, and not a causality. Even so it is interesting that the therapist’s chosen focus have an impact on RTW, also when controlled for the patient’s expectancy of future work ability and actual work ability at the beginning of treatment. This suggests that a work-focused therapy approach is beneficial in achieving RTW. In the uncontrolled analyses also cognitive behavioural interventions, as defined in the TIQI, was significantly associated with RTW. In the TIQI, this factor is constructed from five single items: 1) “Gave patient exercises to practice while away from therapy”, 2) “Used a planned program in the treatment”, 3) “Tried to show that many of the patient’s fears were based on irrational beliefs”, 4) “Used techniques to overcome patient’s symptoms” and 5) “Set strict goals for patient to achieve in therapy” [27]. Of these five, the third is the most typical for CBT, whereas the other four are also important in other structured forms of goal-oriented psychotherapy. This is consistent with studies showing that CBT as well as other structured, goal-oriented therapy approaches are effective in helping people achieve RTW [5, 6]. The treatment modality or number of sessions were not significant predictors of RTW. One interpretation of this finding is that therapists managed to match the treatment with the needs of the individual patient, thereby reducing the predictive power of these variables. Time on a waiting list was not a predictor of RTW in this study. This is contrary to research showing that early interventions support RTW [3].

### Secondary outcome measures and RTW

We also wanted to investigate whether improvements on secondary outcome measures were associated with RTW. Controlled for improved work ability and reduced symptom-score, improved expectancy of future work ability was the only remaining significant predictor of RTW. That improvement in symptoms was not associated with RTW is consistent with the literature. Lagerveld et al. found that work-focused CBT resulted in faster RTW compared with standard CBT, while there was no difference for symptom relief [36].

### Strengths and limitations

A strength of this study is that it included variables relating to both patient characteristics, treatment content and changes during treatment, and information from both patients, therapists and medical records. This made it possible to investigate both demographic and clinical variables as predictors of RTW and to identify the treatment interventions that were associated with RTW. We also used measurements that are routinely used in clinical practise to measure symptoms and functioning. This makes it more likely that our findings have relevance to routine clinical practice. Since this was an uncontrolled pre- post study, changes during treatment could be described, but no conclusions about causality can be drawn. Effect sizes should also be interpreted with some caution.

A weakness in this study is that selection might have introduced a bias in which patients were included. However, we were able to collect some data for the entire population, and for age, gender, GAF scores or diagnoses there were no significant differences between included patients and the whole population. Sixty-one percent of the included patients returned the questionnaire at both pre- and post-treatment, which suggests that self-selection also might have introduced a bias in the final sample. However, analyses showed that there were no significant differences between drop-outs and the final sample, on patient characteristics or mental health status at baseline. Furthermore, there were no significant differences in treatment modality, number of sessions or time on a waiting list. We therefore concluded that the sample did not differ significantly from all included patients for those variables that could be checked.

## Conclusions

This study showed that baseline scores of work ability and expectations of future work ability, having a history of psychiatric treatment and focus on RTW in the treatment predicts RTW among patients in treatment for CMDs. Improvement in the patient’s expectations of future work ability was also associated with actual RTW. These findings can inform future development of RTW interventions for patients with CMDs. Assessing expectations of RTW at the beginning of treatment is recommended to identify persons at risk of future disability. It is essential that treatment is focused not only on symptom-relief, but also on improving work ability and actual RTW. A RTW-focused approach in therapy is associated with RTW. In this study, 6 in 10 patients reported that aspects of their work situation were causing the problems for which they were seeking help. The patient’s working conditions should be assessed as part of treatment, and future research should study working conditions as predictors of RTW among patients with common mental disorders.

## Additional files

Additional file 1: Questionnaire to patients. Questions about socio-demographics, work situation and mental health answered by patients at the beginning and the end of treatment. (ZIP 86 kb)

Additional file 2: Questionnaire to therapists 1. Questions about diagnosis, work situation and treatment history, answered by therapists at the beginning of treatment. (ZIP 38 kb)

Additional file 3: Questionnaire to therapists 2. Questions about mental health status and treatment content, answered by therapists at the end of treatment. (ZIP 43 kb)

## Abbreviations

CMD: Common mental disorder
CORE-OM: Clinical Outcomes in Routine Evaluation Outcome Measure
GP: General practitioner
ICD-10: The International Classification of Diseases – Tenth Edition
RTW: Return to work
SPSS: Statistical Package for the Social Sciences
