# Supplementary material for: Predictors of return to work among patients in treatment for common mental disorders: a pre-post study
Source: BMC Public Health. 2017 Jul 18;18:27. doi: 10.1186/s12889-017-4581-4 (PMC5516307; doi:10.1186/s12889-017-4581-4)
Supplement: Supplementary file 1 — Questionnaire to patients. Questions about socio-demographics, work situation and mental health answered by patients at the beginning and the end of treatment. (ZIP 86 kb) [file 12889_2017_4581_MOESM1_ESM.zip › Additional_file1_EnglishR3.pdf]

This box is to be filled in by the researcher: ID____ Time ___ Date ________

Background information

1. Year of birth (4 digits): ________

2. Gender
1 Male
2 Female

3. Marital status
1 Single
2 Married/civil partner
3 Cohabitant
4 Widow/widower
5 Divorced
6 Separated

4. Do you have children living at home?
1 Yes If yes, state the number of children living at home: ____
2 No

5. Formal education (in number of years)
What is your highest completed education?
(check one box only)
☐ Comprehensive school (1–9 years)
☐ Secondary school or vocational school (10–12 years)
☐ College degree (13–16 years)
☐ Higher university degree (> 16 years)

Labour market participation

6. Occupation (education): ___________________

7. Are you currently working (employed)?
Do not take into consideration whether you are on sick leave or absent from your job for another reason.
1 Yes
2 No **If no, proceed to question 9**

8a. Principal position: __________________ (____%)

8b. Secondary position, if relevant:
_____________________ (____%)

Work and health

9. What is your present work and benefit situation?
(If relevant, check more than one box and enter percentages)

Example 1: I work 50% of a full-time position, and have been on full sick leave. Answer: On 100% sick leave

Example 2: I have an ordinary job and work 50% of a full-time position and receive 50% work assessment allowance. Answer: Ordinary work 50%, work assessment allowance 50%

☐ Ordinary work ___%
☐ Sickness benefit ___%
☐ Active sick leave ___%
☐ Leave of absence from work ___%
☐ Work assessment allowance ___%
☐ Rehabilitation benefit ___%
☐ Rehabilitation ___%
☐ Time-limited disability ___%
☐ Permanent disability benefit ___%
☐ Unemployed ___%
☐ In school ___%
☐ Other ___%

If other, specify:_____________________
(for example via a private pension scheme, KLP, the Norwegian Public Service Pension Fund, welfare benefits etc.)

10. How many full working days have you missed due to health problems (illness, treatment or examinations) in the past three months? _____ days

11. Do you feel that your capacity for work is limited by the problems you are now seeking help for?
1 Yes, definitely
2 Yes, to some degree
3 No, not really
4 No, absolutely not

12. Are there aspects of your work situation that are a direct cause of the problems you are now seeking help for?

1 Yes, definitely
2 Yes, to some degree
3 No, not really
4 No, absolutely not

13. Do you believe, according to your present state of health, that you will be able to do your current job two years from now (or, if you are not currently employed, your previous job)?

1 Yes, definitely
2 Yes, to some degree
3 No, not really
4 No, absolutely not

14. Assume that your work ability at its best has a value of 10 points. How many points would you give to your current work ability? (0 means that you cannot currently work at all.) Circle the number that you feel corresponds to your present work ability.

0	1	2	3	4	5	6	7	8	9	10
Completely unable to work										Work ability at its best
