# Supplementary material for: Predictors of return to work among patients in treatment for common mental disorders: a pre-post study
Source: BMC Public Health. 2017 Jul 18;18:27. doi: 10.1186/s12889-017-4581-4 (PMC5516307; doi:10.1186/s12889-017-4581-4)
Supplement: Supplementary file 1 — Questionnaire to patients. Questions about socio-demographics, work situation and mental health answered by patients at the beginning and the end of treatment. (ZIP 86 kb) [file 12889_2017_4581_MOESM1_ESM.zip › Additional_file1_NorwegianR3.pdf]

Denne rammen fylles ut av forsker: ID____ Tidspunkt ___ Dato ________

Bakgrunnsopplysninger

1.Fødselsår (4 siffer): ________

2. Kjønn
1 Mann
2 Kvinne

3. Sivilstand
1 Enslig
2 Gift/partnerskap
3 Samboer
4 Enke/enkemann
5 Skilt
6 Separert

4.Har du barn som bor hjemme?
1 Ja Hvis, ja, antall barn som bor hjemme: ____
2 Nei

5.Formell utdannelse (i antall år)
Hva er din høyeste fullførte utdanning
(sett bare et kryss)
☐ Grunnskole (1-9 år)
☐ Videregående eller yrkesskole (10-12 år)
☐ Høyskole eller universitet (13-16 år)
☐ Høyere universitetsgrad (> 16 år)

Tilknytning til arbeidslivet

6.Yrke (utdanning): ___________________

7.Har du et arbeidsforhold (ansettelsesforhold) nå? Ikke ta hensyn til om du er sykemeldt er borte fra denne jobben av annen grunn.
1 Ja
2 Nei **Hvis nei, gå direkte til spørsmål 9**

8a.Hovedstilling: _____________________ (____ %)

8b.Ev. bistilling: ______________________ (____ %)

Arbeid og helse

9. Hvordan er din arbeids- og stønadssituasjon i dag?
(Sett eventuelt flere kryss og fyll ut prosentandel)

Eksempel 1: Jeg har 50 % stillingsstørrelse, og har vært fullt sykemeldt. Svar: Sykemeldt 100 %

Eksempel 2: Jeg er 50 % i ordinært arbeid, og 50 % på arbeidsavklaringspenger. Svar: Ordinært arbeid 50 %, arbeidsavklaringspenger 50 %.

☐ Ordinært arbeid ___% andel
☐ Sykepenger ___% andel
☐ Aktiv sykemelding ___% andel
☐ Permisjon fra jobb ___% andel
☐ Arbeidsavklaringspenger ___% andel
☐ Rehabiliteringspenger ___% andel
☐ Attføring ___% andel
☐ Tidsavgrenset uføre ___% andel
☐ Varig uførestønad ___% andel
☐ Arbeidsledig ___% andel
☐ På skole ___% andel
☐ Annet ___% andel

Om annet, spesifiser:___________________________
(for eksempel via privat pensjonsordning, KLP, Statens pensjonskasse, sosial stønad etc.)

10. Hvor mange hele arbeidsdager har du vært borte fra jobben på grunn av helseproblemer (sykdom, behandling eller undersøkelse) i løpet av de siste 3 måneder? _____ dager

11.Opplever du at din arbeidsevne er begrenset av de problem du nå søker hjelp for?
1 Ja, i høy grad
2 Ja, i noen grad
3 Nei, ikke noe særlig
4 Nei, overhodet ikke

12.Er det forhold ved din arbeidssituasjon som er direkte årsak til de problem du søker hjelp for?

1 Ja, i høy grad
2 Ja, i noen grad
3 Nei, ikke noe særlig
4 Nei, overhodet ikke

13.Sett ut fra din helse, tror du at du vil være i stand til å utføre ditt nåværende arbeid om to år (eller ditt tidligere arbeid, om du ikke er i et arbeidsforhold for tiden)?

1 Ja, i høy grad
2 Ja, i noen grad
3 Nei, ikke noe særlig
4 Nei, overhodet ikke

14. Vi går ut fra at din arbeidsevne på sitt beste verdsettes med 10 poeng. Hvor mange poeng vil du gi din nåværende arbeidsevne? (0 betyr at du ikke er i stand til å arbeide for øyeblikket). Sett ring rundt det tallet du mener stemmer men din nåværende arbeidsevne.

	0	1	2	3	4	5	6	7	8	9	10	
Helt uten evne til å arbeide											Arbeids evne på sitt beste	
