# Supplementary material for: Predictors of return to work among patients in treatment for common mental disorders: a pre-post study
Source: BMC Public Health. 2017 Jul 18;18:27. doi: 10.1186/s12889-017-4581-4 (PMC5516307; doi:10.1186/s12889-017-4581-4)
Supplement: Supplementary file 2 — Questionnaire to therapists 1. Questions about diagnosis, work situation and treatment history, answered by therapists at the beginning of treatment. (ZIP 38 kb) [file 12889_2017_4581_MOESM2_ESM.zip › Additional_file2_EnglishR3.pdf]

THERAPIST FORM 1   AFTER FIRST SESSION

To be filled in by the researcher: ID________ Date_________

1. Patient's year of birth:_______________   2. □ Male   □ Female

3. The patient attended   □ Preparatory interview, patient course/group
□ First individual therapy session

4. Has the patient previously received treatment in the mental health care system?
□ No   □ Yes
If yes:
□ Centre for child and adolescent psychiatry
□ Outpatient clinic, community mental health centre
□ Psychologist/psychiatrist in private practice
□ Admitted for inpatient treatment
□ Other

5. Is the patient currently on medication for his/her mental health problems?
□ No   □ Yes
If yes, which type of medication?
□ Antipsychotics
□ Antidepressants
□ Anxiolytics
□ Hypnotics
□ Other

6. The patient is

	Full-time	Part-time
□ Working	□	□
□ On sick leave	□	□
□ Unemployed	□	□
□ Not working		

If the patient has attended a preparatory interview for a patient course/group, only answer questions 7 and 8 if a diagnostic/GAF assessment has actually been carried out.

7. Diagnosis after first session (ICD-10; F or Z code, e.g. F 42.1)
1.____ (Primary diagnosis) □ Preliminary
2. ___________________________________ (Secondary diagnosis, if any) □ Preliminary

8. GAF in the first session

GAF-F (Functioning):____________

GAF-S (Symptoms):____________

THANK YOU VERY MUCH FOR YOUR HELP.
