# Supplementary material for: Predictors of return to work among patients in treatment for common mental disorders: a pre-post study
Source: BMC Public Health. 2017 Jul 18;18:27. doi: 10.1186/s12889-017-4581-4 (PMC5516307; doi:10.1186/s12889-017-4581-4)
Supplement: Supplementary file 2 — Questionnaire to therapists 1. Questions about diagnosis, work situation and treatment history, answered by therapists at the beginning of treatment. (ZIP 38 kb) [file 12889_2017_4581_MOESM2_ESM.zip › Additional_file2_NorwegianR3.pdf]

BEHANDLERSKJEMA 1 ETTER FØRSTE SAMTALE

Fylles ut av forsker: ID________ Dato_________

1. Pasientens fødselsår:_______________ 2. ☐ Mann ☐ Kvinne

3. Pasienten møtte til ☐ Forsamtale pasientkurs/gruppe ☐ Første individualterapitime

4. Har pasienten gått i behandling tidligere i psykisk helsevern?

☐ Nei ☐ Ja

Hvis ja:

☐ BUP

☐ Poliklinikk, DPS

☐ Privatpraktiserende psykolog/psykiater

☐ Innlagt døgnavdeling

☐ Annet

5. Får pasienten for tiden medikamenter for sine psykiske problemer?

☐ Nei ☐ Ja

Hvis ja, hvilken type medikament?

☐ Antipsykotika

☐ Antidepressiva

☐ Angstdempende

☐ Sovemedisin

☐ Annet

6. Pasienten er

	Heltid	Deltid
☐ I arbeid	☐	☐
☐ Sykemeldt	☐	☐
☐ Arbeidsledig	☐	☐
☐ Ikke i arbeid		

Hvis pasienten har møtt til forsamtale for pasientkurs/gruppe besvares spørsmål 7 og 8 kun hvis diagnose/GAF faktisk er blitt vurdert.

7. Diagnose etter første samtale (ICD-10; F eller Z kode, for eksempel F 42.1)

1.____ (Hoveddiagnose) ☐ Foreløpig

2. ____ (Evt. bidiagnose) ☐ Foreløpig

8. GAF i første samtalen

GAF-F (Funksjon):___________

GAF-S (Symptom):___________

STOR TAKK FOR HJELPEN!
