# Supplementary material for: Predictors of return to work among patients in treatment for common mental disorders: a pre-post study
Source: BMC Public Health. 2017 Jul 18;18:27. doi: 10.1186/s12889-017-4581-4 (PMC5516307; doi:10.1186/s12889-017-4581-4)
Supplement: Supplementary file 3 — Questionnaire to therapists 2. Questions about mental health status and treatment content, answered by therapists at the end of treatment. (ZIP 43 kb) [file 12889_2017_4581_MOESM3_ESM.zip › Additional_file3_EnglishR3.pdf]

THERAPIST FORM 2 CONCLUSION OF THERAPY

To be filled in by the researcher: ID________ Date_________

1. Patient's year of birth:______________ 2. □ Male □ Female

3. Has the patient undergone individual therapy? □ No □ Yes
If yes, number of sessions (indiv. therapy):_____________

4. Has the patient attended (or will he/she be attending) a patient course? □ No □ Yes

NOTE! If the patient has only attended a patient course, the course leader does not have to fill in the rest of the form (from question 5).

5. To what extent has the therapy focused on the patient returning to work (or, for patients who have not been on sick leave, on continuing to work)?
Circle the alternative you deem most suitable

Not at all 1 2 3 4 5 6 7 8 9 **Very much**

6. The patient is

	Full-time	Part-time
□ Working	□	□
□ On sick leave	□	□
□ Unemployed	□	□
□ Not working		

7. Is the patient currently on medication for his/her mental health problems?
□ No □ Yes
If yes, which type of medication?
□ Antipsychotics
□ Antidepressants
□ Anxiolytics
□ Hypnotics
□ Other

8. Diagnostic assessment on completion of treatment (ICD-10; F or Z code, e.g. F 42.1)
The diagnosis/diagnoses that best describe(s) the condition the patient has received treatment for.
1.____ (Primary diagnosis) □ Tentative
2. _______________________________________ (Secondary diagnosis, if any) □ Tentative

9. GAF on completion of treatment (final contact)

GAF-F (Functioning):___________

GAF-S (Symptoms):___________

THANK YOU VERY MUCH FOR YOUR HELP.
