# Supplementary material for: Predictors of return to work among patients in treatment for common mental disorders: a pre-post study
Source: BMC Public Health. 2017 Jul 18;18:27. doi: 10.1186/s12889-017-4581-4 (PMC5516307; doi:10.1186/s12889-017-4581-4)
Supplement: Supplementary file 3 — Questionnaire to therapists 2. Questions about mental health status and treatment content, answered by therapists at the end of treatment. (ZIP 43 kb) [file 12889_2017_4581_MOESM3_ESM.zip › Additional_file3_NorwegianR3.pdf]

BEHANDLERSKJEMA 2 AVSLUTNING AV TERAPI

Fylles ut av forsker: ID________ Dato_________

1. Pasientens fødselsår:_______________ 2. ☐ Mann ☐ Kvinne

3. Har pasienten gått i individualterapi? ☐ Nei ☐ Ja
Hvis ja, antall konsultasjoner (ind. terapi):______________

4. Har pasienten gått (eller skal gå på) pasientkurs? ☐ Nei ☐ Ja

NB! Hvis pasienten kun har gått pasientkurs trenger kursholder ikke å fylle ut resten av skjemaet (fra spm. 5 og videre).

5. I hvor stor grad har terapien fokusert på pasientens tilbakegang til arbeid (eller det å stå i arbeid for pasienter som ikke har vært sykemeldt)?
Sett en ring rundt det trinn som du mener passer best

**Ikke i det hele tatt**	1	2	3	4	5	6	7	8	9	**I svært stor grad**

6. Pasienten er

	Heltid	Deltid
☐ I arbeid	☐	☐
☐ Sykemeldt	☐	☐
☐ Arbeidsledig	☐	☐
☐ Ikke i arbeid		

7. Får pasienten for tiden medikamenter for sine psykiske problemer?

☐ Nei ☐ Ja

Hvis ja, hvilken type medikament?

☐ Antipsykotika

☐ Antidepressiva

☐ Angstdempende

☐ Sovemedisin

☐ Annet

8. Diagnostisk vurdering ved avslutning (ICD-10; F eller Z kode, for eksempel F 42.1)
Den/de diagnoser som best beskriver hva tilstand pasienten har gått i behandling for.

1.____ (Hoveddiagnose) ☐ Tentativ

2. ____ (Evt. bidiagnose) ☐ Tentativ

9. GAF ved avslutning (ved siste kontakt)

GAF-F (Funksjon):____________

GAF-S (Symptom):____________

STOR TAKK FOR HJELPEN!
